# NG2-positive pericytes regulate homeostatic maintenance of slow-type skeletal muscle with rapid myonuclear turnover

**DOI:** 10.1186/s13287-023-03433-1

**Published:** 2023-08-17

**Authors:** Takamitsu Tatsukawa, Kohei Kano, Kei-ichi Nakajima, Takashi Yazawa, Ryoji Eguchi, Maki Kabara, Kiwamu Horiuchi, Taiki Hayasaka, Risa Matsuo, Naoyuki Hasebe, Nobuyoshi Azuma, Jun-ichi Kawabe

**Affiliations:** 1 Department of Biochemistry, Asahikawa Medical University, 2-1-1 Midorigaoka-Higashi, Asahikawa, 078-8510 Japan; 2 Department of Vascular Surgery, Asahikawa Medical University, 2-1-1 Midorigaoka-Higashi, Asahikawa, 078-8510 Japan; 3 Department of Cardiovascular Regeneration and Innovation, Asahikawa Medical University, 2-1-1 Midorigaoka-Higashi, Asahikawa, 078-8510 Japan; 4 Division of Cardiovascular, Respiratory and Neurology, Department of Medicine, Asahikawa Medical University, 2-1-1 Midorigaoka-Higashi, Asahikawa, 078-8510 Japan; 5 Department of Dermatology, Asahikawa Medical University, 2-1-1 Midorigaoka-Higashi, Asahikawa, 078-8510 Japan

**Keywords:** Myogenesis, Skeletal muscle, Slow-type muscle, Stem cells, Pericytes

## Abstract

**Background:**

Skeletal muscle comprises almost 40% of the human body and is essential for movement, structural support and metabolic homeostasis. Size of multinuclear skeletal muscle is stably maintained under steady conditions with the sporadic fusion of newly produced myocytes to compensate for the muscular turnover caused by daily wear and tear. It is becoming clear that microvascular pericytes (PCs) exhibit myogenic activity. However, whether PCs act as myogenic stem cells for the homeostatic maintenance of skeletal muscles during adulthood remains uncertain.

**Methods:**

We utilized PC-fused myofibers using PC-specific lineage tracing mouse (NG2-CreERT/Rosa-tdTomato) to observe whether muscle resident PCs have myogenic potential during daily life. Genetic PC deletion mouse model (NG2-CreERT/DTA) was used to test whether PC differentiates to myofibers for maintenance of muscle structure and function under homeostatic condition.

**Results:**

Under steady breeding conditions, tdTomato-expressing PCs were infused into myofibers, and subsequently, PC-derived nuclei were incorporated into myofibers. Especially in type-I slow-type myofibers such as the soleus, tdTomato^+^ myofibers were already observed 3 days after PC labeling; their ratio reached a peak (approximately 80%) within 1 month and was maintained for more than 1 year. Consistently, the NG2^+^ PC-specific deletion induced muscular atrophy in a slow-type myofiber-specific manner under steady breeding conditions. The number of myonucleus per volume of each myofiber was constant during observation period.

**Conclusions:**

These findings demonstrate that the turnover of myonuclei in slow-type myofibers is relatively fast, with PCs acting as myogenic stem cells—the suppliers of new myonuclei under steady conditions—and play a vital role in the homeostatic maintenance of slow-type muscles.

**Supplementary Information:**

The online version contains supplementary material available at 10.1186/s13287-023-03433-1.

## Introduction

Dynamic cellular equilibrium, a balance between gaining and losing cells, is an essential characteristic of multicellular organisms and strictly regulated for adaptation to variety of their external and internal conditions including activity, anabolism/catabolism, and diseases. In contrast to many mononuclear cells, skeletal muscle cells are one of the few syncytial cells. According to the myonuclear domain theory, a given myonucleus has limited transcriptional capacity and controls a defined volume of the myocytoplasm. Based on this theory, a linear relationship exists between the total number of myonuclei and muscle fiber size [1, 2]. The myonuclear number is adjusted to maintain the proper nuclear-to-cytoplasmic ratio—new nuclei are added during hypertrophy and lost with atrophy. Thus, the number of myonuclei is altered accordingly during muscle hypertrophy and atrophy [3]. However, the mechanisms by which the myonuclei are turned over, and skeletal muscles are maintained during adulthood remain unclear.

Since skeletal muscle nuclei are post-mitotic, the remarkable muscular regenerative and homeostatic maintenance potencies are owned by the resident myogenic stem cell population, called satellite cells (SCs). These cells fuse into the syncytium for myonuclear addition or replacement [4–6]. Recent studies utilizing conditional SC-deletion mouse models have reported that effective myofiber hypertrophy occurs in the absence of SCs [7]. SCs are not globally required to maintain muscle mass throughout the lifespan [8, 9]. Therefore, myonuclei are assumed to be supplied by SCs and other myogenic cells to sustain muscle mass under homeostatic conditions.

Pericytes (PCs) are mural cells embedded in the capillary basal lamina that regulate fundamental microvessel functions, such as blood flow and permeability [10, 11]. Some PC populations exhibit multipotency, similar to mesenchymal stem cells (MSCs) [12–15]. Several reports indicate that PCs, which are distinct from SCs, can differentiate into skeletal muscles in vitro and in vivo [14, 16, 17]. Experiments using lineage tracing of alkaline phosphatase^+^ PCs revealed that PCs fuse with developing myofibers and become Pax7^+^ SCs, contributing to myogenic growth during postnatal muscular development [18]. Furthermore, Kostallari et al. [19] reported that PCs are indispensable for postnatal skeletal muscle growth using a transgenic mouse model for the selective deletion of neuroglial 2 proteoglycan (NG2)^+^ PCs. PCs stimulate muscle growth through insulin-like growth factor 1 and regulate SC quiescence through angiopoietin, subsequently promoting muscle growth during the neonatal period. However, the myogenic effects of PCs, through myogenic stem cells and/or SC-associated cells, are restricted to the neonatal/juvenile developmental stage. However, the role of PCs in muscular maintenance during adulthood is not well understood.

Besides PCs, fibro-adipogenic progenitors (FAPs) have various functions, such as the maintenance of somatic stem cells, including SCs [20, 21], and their MSC-like multipotency contributes to ectopic fatty formation in skeletal muscle tissues [22]. Platelet-derived growth factor receptor alpha (PDGFRα)^+^ FAPs are reportedly required for the homeostatic maintenance of adult skeletal muscle by providing an SC-sustaining microenvironment [23, 24]. Additionally, the inducible depletion of FAPs in adult mice under normal breeding conditions for up to 9 months reduced the number of SCs and resulted in muscle atrophy [23]. Further, Uezumi et al. [24] reported that inducible FAP-deletion exhibits phenotypes markedly similar to sarcopenia, including myofiber atrophy, alterations in fiber types, and denervation at neuromuscular junctions. However, the inability to genetically target FAPs in vivo has limited the accurate assessment of their roles in muscle regeneration and homeostasis. Ultimately, whether myogenic stem cells other than SCs act on the homeostatic maintenance of skeletal muscles during adulthood remains uncertain.

In this study, PC-specific lineage tracing and inducible PC-deletion mouse models were used to address the necessity of PC in adult skeletal muscle maintenance. PC-specific lineage tracing experiments were performed to demonstrate the myonuclear turnover and the contribution of PCs to myonuclear supplementation. Collectively, we aimed to elucidate the role of microvascular PCs in maintaining long-term homeostasis in the skeletal muscles.

## Materials and methods

### Animals

All mice used in this study had a C57BL/6 J genetic background and were housed under specific pathogen-free (SPF) conditions and kept at 22–26 °C under 12 h:12 h light–dark cycle and provide regular chow ad libitum and tap water during the experiment. The NG2-specific fluorescent mice (*NG2*-DsRed mice) and NG2-specific Cre-inducible cell lineage tracing mice (*NG2-CreERT*/*Rosa26-STOP-*floxed tdTomato-Tg [NG2-CreERT/Rosa TdTomato] mice; The Jackson Laboratory) were generated as previously described [25, 26]. The NG2-specific Cre-inducible cell deletion mice (NG2-CreERT/DTA mice) were generated by crossing NG2-CreER mice and *Rosa26*-STOP-floxed-DTA-Tg mice (The Jackson Laboratory) [27]. Male mice [12–16 weeks of age, 25 ± 5 g body weight (bw)] were used for all experiments. To induce Cre recombinase, mice were treated with Tam (Tamoxifen; Sigma-Aldrich, St. Louis, MO, USA) intraperitoneally at a dose of 100 mg/kg bw for 5 days. For long-term observation (more than 1 month), additional Tam (100 mg/kg bw) was injected monthly to maintain PC deletion. *Rosa26*-STOP-floxed-DTA mice treated with Tam and NG2-CreERT/DTA mice treated with vehicle and corn oil were regarded as controls A and B, respectively. All animal experiments were performed in accordance with the ethical guidelines approved by the Animal Care and Use Committee of Asahikawa Medical University.

### Physiological performance tests

Muscle strength was assessed using a grip-strength device (MK-380 M; Muromachi Kikai Co., Tokyo, Japan). Mice were held by the tail and were made to grab the wire mesh of the grip-strength device with each limb. The mice were gently pulled away until the grip was released, and the maximal force was recorded. Ten measurements were performed 10 times for each mouse. Exercise tolerance was assessed using a treadmill test, with minor modifications to a published protocol [17]. Briefly, the mice were placed on the belt of a lane motorized treadmill (TMS-4B; MELQUEST, Toyama, Japan). After a warm-up period of 5 min (flat lane, belt speed of 10 m/min), the mice were run under the test conditions (+ 15° slope lane, 15 m/min), and the maximum running time was measured.

### Histology and immunohistochemistry

To estimate functional vessels, mice were anesthetized with isoflurane (between 1.5 and 2.5%) to minimize suffering and injected with 300 µL of fluorescein isothiocyanate (FITC)- or rhodamine-labeled Griffonia simplicifolia lectin (500 µg/mL in PBS; Vector Laboratories, Burlingame, USA) before euthanasia, as described previously [25]. Euthanasia was performed by cardiac puncture under isoflurane anesthesia. Fresh muscle samples were embedded in a compound (Surgipath FSC 22 Blue; Leica Biosystems, Wetzlar, Germany), quickly frozen in liquid nitrogen, and stored at − 80 °C until further use.

For histological analyses, 12-µm sections were obtained from frozen muscle using a cryostat (CM3050S; Leica Biosystems, Wetzlar, Germany) and fixed with 4% paraformaldehyde (PFA) in PBS (pH 7.0) for 5 min. Hematoxylin–eosin staining of the sections was performed using standard methods. Immunofluorescence analyses were conducted as previously described [17, 25]. Briefly, freshly frozen sections were fixed with acetone for 5 min at − 30 °C. After air-drying, the samples were incubated with 0.3% Triton X-100 in PBS (PBST) with blocking buffer (1% bovine serum albumin [BSA] in PBST) for 60 min. Primary antibodies used were anti-myosin heavy chain type I (BA-D5-s, mouse monoclonal IgG2b, 1:10, DSHB, Iowa, USA), type IIA (SC-71-s, mouse monoclonal IgG1, 1:10, DSHB), type IIB (BF-F3-s, mouse monoclonal IgM, 1:10, DSHB), and anti-fast myosin skeletal heavy chain (ab91506, rabbit polyclonal, 1:100, abcam). Bound antibodies were visualized using following secondary antibodies: DyLight405-conjugated anti-mouse IgG 2b subclass-specific (115-475-207, goat polyclonal, 1:1000, Jackson, West Grove, PA, USA), specifically detect BA-D5, Alexa488-conjugated anti-mouse IgG1 subclass-specific (115-545-205, goat polyclonal, 1:1000, Jackson), specifically detect SC-71, Alexa647-conjugated anti-mouse IgM subclass-specific (ab150123,goat polyclonal, 1:1000, abcam), specifically detect BF-F3, and Alexa 488-conjugated anti-rabbit IgG (A11008,goat polyclonal, 1:1000, invitrogen). Nuclei were stained with 4ʹ, 6-diamidino-2-phenylindole (DAPI; H-1200, VECTOR, CA, USA). Images were obtained using a fluorescence microscope (BZ-X710; Keyence).

To observe myofibers with microvessels in a 3D view, 50 µm longitudinal sections were fixed with 4% PFA and transparency using RapiClear 1.47 (RC147001, SunJin, Taiwan) was performed as described previously [25]. Clarified tissue sections were imaged using a confocal fluorescence microscope (LMS900; Carl Zeiss, Oberkochen, Germany), and 20–30 serial slides (40 ×) in 2-µm steps were z-stacked and projected onto a 3D image. Image analysis was performed using the BZ-X Analyzer (Keyence), ZEN blue (Carl Zeiss), and ImageJ software (ver.1.53c, National Institutes of Health, Maryland, USA).

### Measurement of myonuclear domain

Isolation of single myofibers was performed with modifications to the previous description　[28]. Briefly, after skeletal muscles were fixed with 4% PFA for 48 h, the muscles were incubated in a 40% NaOH solution for 2 h at 24 °C to isolate single myofibers. Muscle samples were then neutralized by soaking in 1 M Tris HCL solution (pH 6.0). The isolated myofibers were mounted on glass slides with 10% glycerol containing Hoechst 33,342 (H3570; Invitrogen, Thermo Fisher Scientific, Waltham, MO, USA). Isolated myofibers were imaged using a fluorescence microscope (BZ-X710; Keyence), and the area of each myofiber and the number of nuclei within the fibers were measured.

### *Fluorescence *in situ* hybridization (FISH)*

To detect NG2^+^ PC-originated myonuclei, the nuclei in which genetic recombination was induced by NG2 Cre were detected using PCR-FISH methods. To detect the Cre-specific genetic recombination site (tdTomato gene), the following primers were designed: forward primer, GGGCCCTAAGAAGTTCCTATTC; reverse primer, GGGGAAGGACAGCTTCTTGT. Myofibers were labeled by in situ PCR to synthesize digoxigenin (DIG)-labeled DNA using the PCR DIG Probe Synthesis Kit (Sigma-Aldrich, St. Louis, MO, USA) according to the manufacturer’s protocol, with some modifications (denaturation at 95 °C for 40 s, annealing at 60 °C for 20 s, and elongation at 72 °C for 15 s). DIG-labeled probes were detected with an anti-DIG antibody (ab420, mouse monoclonal, 1:100, Abcam) and visualized with Alexa 488-conjugated anti-mouse IgG antibody (A11029, goat polyclonal, 1:1000, Invitrogen).

### In vitro* myogenesis assay*

After treatment with Tam for one week, soleus muscle fibers of NG2-lineage mice were isolated using collagenase I solution (100 mg/mL Hank’s PBS). Muscle fiber samples were incubated in a complete Dulbecco’s modified Eagle’s medium (DMEM; Gibco, Thermo Fisher Scientific, Waltham, MO, USA) containing 20% fetal bovine serum (FBS; CORNING, Corning, NY), 100 U/mL penicillin, and 100 μg/mL streptomycin. After the cells were grown from fiber explants, the medium was switched to a differentiation medium—DMEM containing 2% horse serum (Gibco, Thermo Fisher Scientific, Waltham, MO, USA). After changing the medium every 4 days, myogenic differentiation was confirmed by observing myotube formation or immunostaining of MyHC, MYH2 and MYH7.

### In vitro* cell viability assay*

Adipose stromal cells (ASCs) were prepared from subcutaneous adipose tissues of the NG2CreERT/ DTA mice, and NG2^+^cells were isolated using magnetic-activated cell sorting system as described previously [29]. Isolated cells (1.5 × 10^4^ cells per well) were seeded on 12-well plates and incubated overnight and then, treated with 2 µM 4-hydroxytamoxifen (4-HT; Sigma-Aldrich) or vehicle (DMEM with 10% FBS, 100 U/ml penicillin, and 100 mg/ml streptomycin) for 6 days. Cell numbers were counted in four high-power fields for each well, and the averages were compared.

### Quantitative reverse-transcription (RT)-PCR

Total RNA was isolated from the soleus muscle or cultured cell samples using TRIzol™ Reagents (Thermo Fisher Scientific, Waltham, MO, USA). Complementary DNA was synthesized using the iScript cDNA Synthesis Kit (Bio-Rad, Hercules, CA, USA). Real-time quantitative RT-PCR was performed in triplicate using TaqMan Gene Expression Master Mix (Thermo Fisher Scientific) on a LightCycler 480 System (Roche Diagnostics, Basel, Switzerland). The following fluorogenic probes and primers were used for detection: Mm99999915_g1 (*Gapdh*), Mm00507257_m1 (*Cspg4*/ *NG2*), Mm01354484_m1 (*Pax7*), Mm00435125_m1(Myf5), Mm00440387_m1 (*Myod*), Mm00446194_m1 (*Myogenin*), Mm01332564_m1 (*Myh2*), Mm01332541_m1 (*Myh4*), and Mm00600555_m1 (*Myh7*). The relative mRNA expression levels were calculated using the comparative threshold cycle method. Glyceraldehyde-3-phosphate dehydrogenase (*GAPDH*) was used as the internal control.

### Gene microarray analysis

One month after treatment with Tam or the vehicle (corn oil), soleus muscles from each group of PC-deletion mice were dissected, frozen in liquid nitrogen, and stored at − 80 °C. The mRNA of these samples was subjected to microarray analysis using the 3D Gene Mouse Oligo Chip 24 K (Toray Industries Inc., Tokyo, Japan), as described previously [15]. The signal corresponding to each gene was normalized using the global normalization method (Cy3/Cy5 ratio median = 1). Intensity values greater than two standard deviations above the background signal were considered valid. GO enrichment analysis was conducted using the metascape.org website.

### Statistical analysis

Experimental data are presented as means ± standard error of the mean (SEM) unless otherwise noted. The sample numbers (n) are shown in the figure legends. Differences between two measurements were evaluated using the unpaired Student’s t-test, and for comparisons of more than two groups, one-way ANOVA was used for normal distributions. This was followed by Tukey’s post hoc test using Prism software version 9.0 (GraphPad, San Diego, CA, USA). A *P* value < 0.05 was considered statistically significant.

## Results

### ***NG2***^+^***PCs contribute to muscular regeneration in a muscle-type-dependent manner during adulthood***

To examine whether PC contributes to myogenesis during in vivo homeostatic conditions, we genetically labeled PCs and observed their long-term fate in uninjured normal adult muscles. We utilized NG2 (neuron-glial antigen 2) as a PC marker since we previously confirmed that PCs were labeled specifically in peripheral tissues, including skeletal muscle (Additional file 1: Fig S1). We efficiently induced recombination in over 80% of NG2^+^ PCs after treatment with tamoxifen (Tam) using an NG2 promoter-derived gene recombination system, NG2-CreERT [17, 25, 29].

NG2-CreERT/Rosa-tdTomato mice were treated with Tam for one week. Muscles were harvested at the indicated time points (Additional file 1: Fig S2). After Tam treatment, tdTomato-expressing cells were observed specifically in PCs adjacent to microvessels in skeletal muscle (Additional file 1: Fig S2). After normal breeding for 1–12 months, tdTomato-expressing cells were observed in myofibers. Notably, the degree of PC contribution to myofibers was different among the muscle types; the soleus and gastrocnemius (limb muscles) comprised 85% and 35% tdTomato^+^ myofibers, respectively, whereas the muscles of the diaphragm and abdominal walls contained 80% and 30%, respectively (Additional file 1: Fig S2). The soleus and diaphragm are rich in slow-type myofibers.

**Fig. 1:**
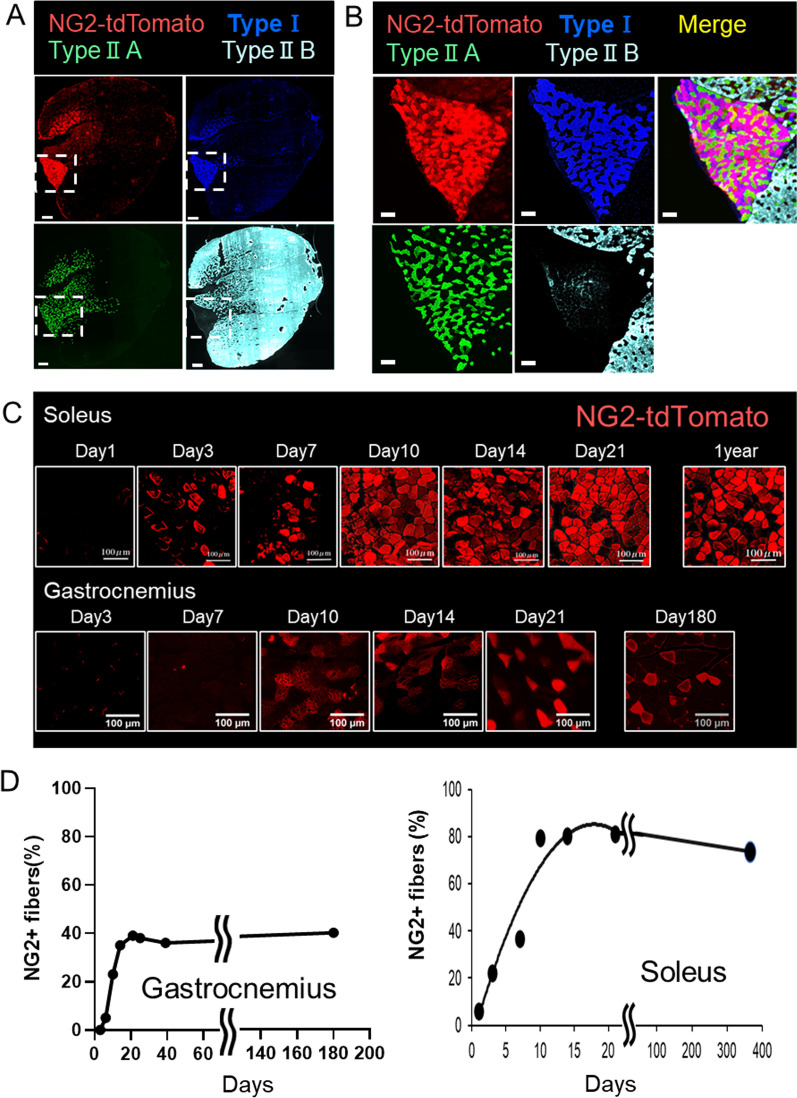
NG2^+^ cell-originated muscle fibers in a muscle-type dependent manner. **A** NG2^+^ PC-specific lineage tracing was performed using NG2-CreERT/Rosa-tdTomato mice. Immunostaining short axis view of lower leg muscle (gastrocnemius and soleus) demonstrates the distribution of each type of muscular fiber, types I, IIA, and IIB. A dashed line square area indicates the soleus. Scale bar = 500 µm. **B** High-power immunostaining view of the soleus area. Scale bar = 200 µm. **C** The representative fluorescence view of tdTomato^+^ myofibers within the soleus and gastrocnemius is shown at the indicated time labeling of NG2^+^ PCs. **D** The time course of the proportion of labeled myofibers to total myofibers is shown. The values are presented as the means ± standard error of the mean (SEM); *n* = 3–4

Skeletal muscle fibers are broadly classified into type I (slow-twitch/fatigue-resistant), type IIB (fast-twitch/fatigue-susceptible), and type IIA (intermediate) based on their physiological properties and myosin heavy chain (MyHC) isoforms [30]. To confirm the muscle-type specificity of PC-contributing myofibers, myofibers were immunolabeled with MyHC isoforms. The soleus muscles contained mostly slow-type myofibers (45, 55, and 5% of type I, IIA, and IIB, respectively), and tdTomato-expressing cells were located in slow-type myofibers 6 months after Tam treatment (Fig. 1A, B). These data suggested that PCs contribute to the selective regeneration of muscles in a slow-type myofiber subset under static breeding conditions.

### ***Rapid turnover of muscular nuclei with NG2***^+^***PCs for maintenance of muscle mass***

Next, we examined the time course of tdTomato labeling in myofibers. The tdTomato-expressing myofibers were observed during the normal breeding period of one week, and their ratio to total myofibers increased and peaked around 1 month after Tam treatment (Fig. 1C, D). Notably, NG2^+^ PCs were labeled with Tam treatment once at the beginning of the experiment, and the peak ratio was maintained for up to 12 months (Fig. 1C, D, Additional file 1: Fig S2).

**Fig. 2:**
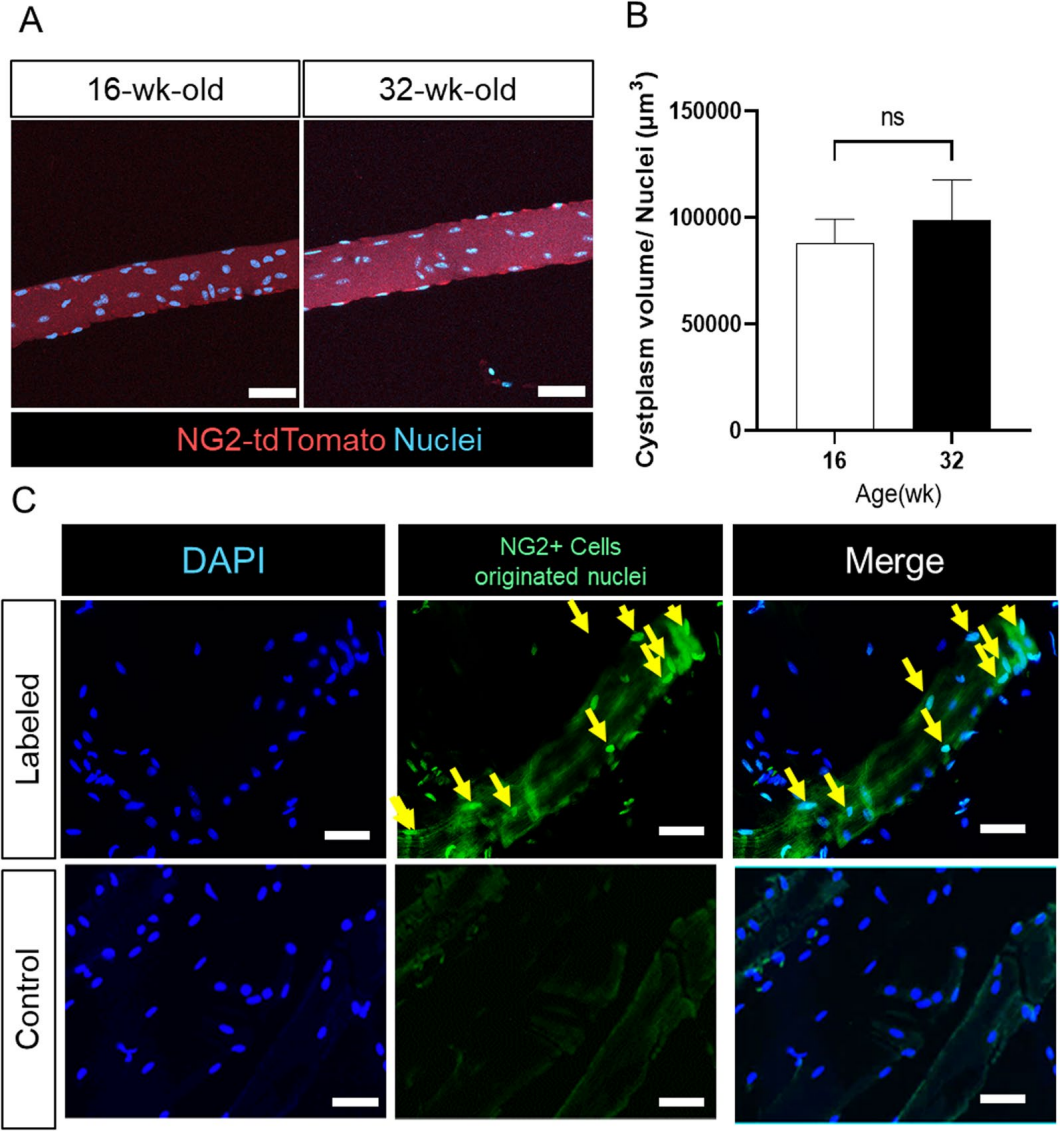
Determination of myonuclei originated from NG2^+^ PCs. **A** Myofibers isolated from the soleus, measured area of each myofiber, and the number of myonuclei. **B** The calculated ratio of the myonuclear number to that of myofibers. The values are presented as mean ± SEM (*n* = 3). ns = not significant. **C**. NG2^+^ PC-originated nuclei within myofibers, determined by fluorescence in situ hybridization (FISH). A non-specific DNA probe was used as a negative control. Nuclei are stained by Hoechst 33,342. Scale bar = 50 µm

Rodent muscle mass is dramatically increased during the postnatal growth period (0–10 weeks old) but remains mostly constant after the growth period under normal breeding conditions. According to the myonuclear domain theory, the myonuclear number in myofibers is stable under a fixed volume of myofibers in adulthood, especially under normal breeding conditions [1]. Indeed, the number of myonuclei in each myofiber of the soleus among the 16–32 week-old mice was constant (Fig. 2A, B).

Because skeletal muscle fibers are multinuclear cells, tdTomato-expressing myofibers can be detected if any myonuclei are replaced with nuclei from NG2^+^ PCs. To detect myonuclei originating from NG2^+^ PC within myofibers, the recombinant tdTomato gene within myonuclei of isolated myofibers was determined using fluorescence in situ hybridization (FISH). NG2^+^ PC-originated nuclei were detected in isolated myofibers of the soleus 2 months after induction of tdTomato expression in NG2^+^ PCs (Fig. 2C). According to the time course of the ratio of tdTomato-labeled myofibers (Fig. 1C, D), PCs contributed to myonuclear replacement (at least one nucleus in each myofiber) at a ratio of 5–7% and 1–2% per day in the soleus and gastrocnemius muscles, respectively. These data suggest that PCs contribute dynamically and persistently to myofiber regeneration through myonuclear replacement under homeostatic conditions.

### ***Muscle-resident NG2***^+^***PCs have myogenic potential to differentiate into muscle fibers***

**Fig. 3:**
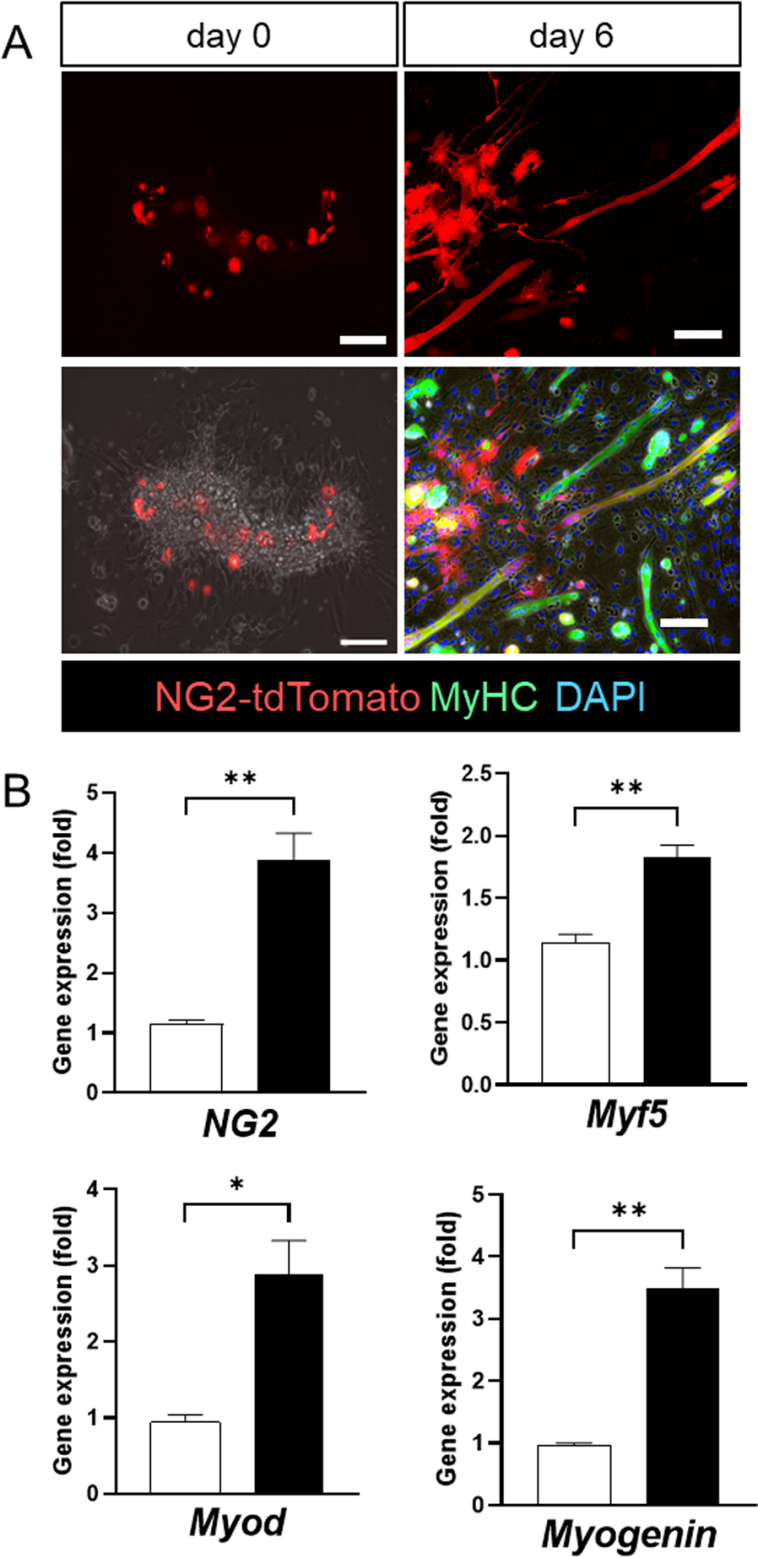
In vitro myogenic potency of NG2^+^ PCs attached around myofibers. **A** Myofibers isolated from the soleus of NG2-CreERT/Rosa-tdTomato mice, which were incubated in DMEM-containing hydroxy tamoxifen (Tam) for 3 days to label NG2^+^ PCs. After 6 days of differentiation induction, myogenesis was determined by immunostaining with myosin heavy chain (MyHC). Nuclei are counterstained by DAPI. Scale bars = 100 µm. **B** Gene expression of myogenesis-related genes in each sample, determined by quantitative RT-PCR analysis. Open bars = differentiation induction (–), and closed bars = differentiation induction ( +). The values are presented as mean ± SEM (*n* = 4). *P < 0.05, **P < 0.01

Other investigators and we have reported that PCs isolated from peripheral tissues, including skeletal muscle and adipose tissue, have myogenic potential in vitro and differentiate into functional skeletal myofibers when transplanted into dystrophy model mice [16, 17]. To minimize artificial modification by cell subculture manipulation, we prepared myofiber explants from skeletal muscles and examined the myogenic potency of primary PCs in the microvessels that attach to myofiber explants (Additional file 1: Fig S3). After 6 days of myogenesis induction, tdTomato^+^ PCs differentiated into myosin heavy chain (MyHC)-stained myofibers (Fig. 3A). In parallel with myogenesis, the expression of myogenesis-related genes, including myoD and myogenin, was increased (Fig. 3B). To test whether myogenic PCs would differentiate to the fiber type similar to where they originated, isolated PCs from soleus muscles differentiate to MyHC-positive myotubes but not to advanced type specific myofibers (Additional file 1: Fig S4).

### ***Deletion of NG2***^+^***PCs induced muscular atrophy, specifically in slow-type muscle fibers***

Although lineage-tracing experiments suggest that PCs contribute to myogenesis through myonuclear replacement, it is possible that PCs fuse into myofibers nonspecifically and that PC-originated nuclei do not act as myonuclei. Thus, we examined the consequences of genetic deletion of PC on the structure and function of skeletal muscle under homeostatic conditions. NG2-CreERT/DTA mice were treated with Tam and subjected to muscular functional and histological analyses at the indicated normal breeding time following PC deletion. NG2CreERT^–/–^/DTA with Tam and NG2-CreERT/DTA without Tam were used as controls A and B, respectively.

**Fig. 4:**
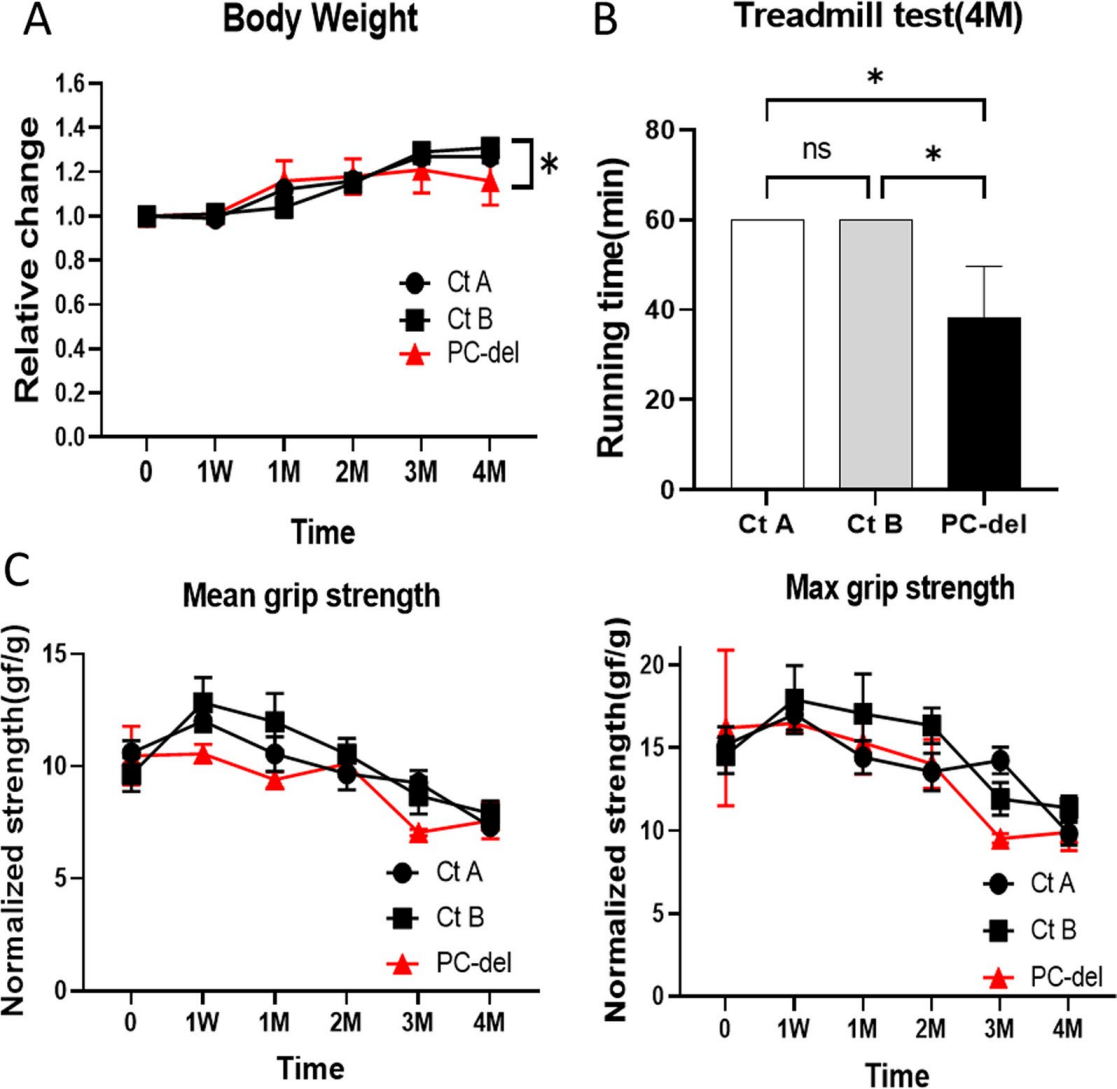
Physiological performance after deletion of NG2^+^ PCs. PC-deletion induced by Tam treatment of NG2-CreERT/Rosa-DTA mice. Rosa-DTA mice with Tam or NG2-CreERT/DTA without Tam were used as control A and B (Ct A, Ct B), respectively. After primary Tam treatment at the start point, mice were treated with Tam every month. **A** The time course of body weight of PC-deletion (PC-del) and control mice. **B** At 4 months after PC deletion, exercise tolerance was assessed using the treadmill test. **C** At indicated time after induction of PC-deletion, muscular power was estimated by the grip strength of four limbs. The value was normalized by body weight, and presented as the means ± SEM (*n *= 4–8); *P < 0.05, ns = not significant

We confirmed that the recombinant CreERT/DTA system appropriately induced deletion of NG2^+^PCs with tamoxifen treatment in vitro system (Additional file 1: Fig S5). However, in vivo general phenotypic changes including circulation disorder and loss of body weight were not observed in the early stages of PC deletion, similar to a previous study [19]. Four months after PC deletion, body weight slightly decreased (Fig. 4A), and the general physiological performance (measured by the treadmill test) was significantly reduced (Fig. 4B). The relative pure muscle strength (tested by grip power) was not altered during the observation period up to 4 months (Fig. 4C). Notably, there was no alteration in body weight, muscular atrophy, and performance in all control groups due to non-specific side effects.

**Fig. 5:**
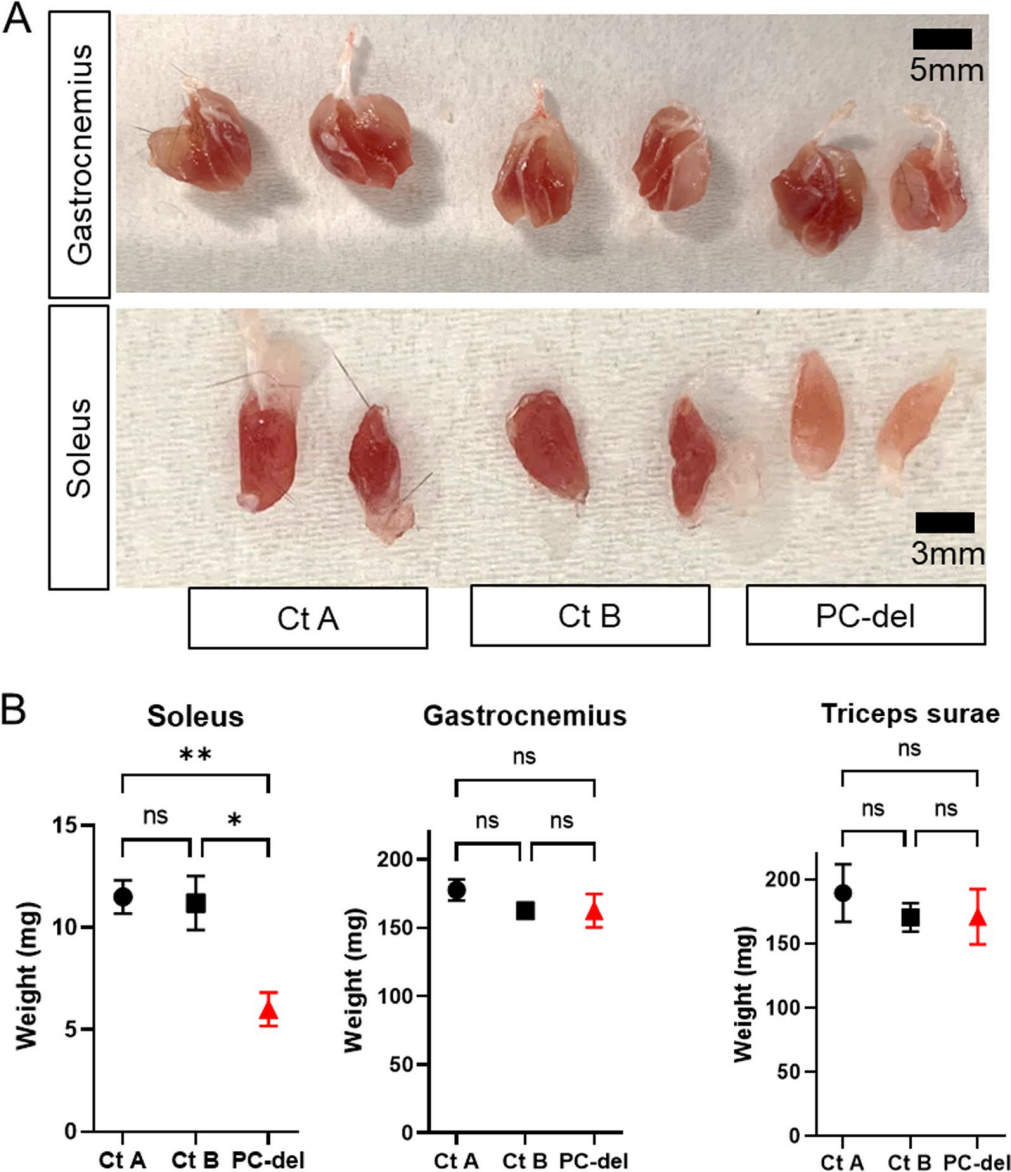
Deletion of NG2^+^ PCs induces muscular atrophy in soleus muscles.** A** Appearance of isolated soleus, gastrocnemius, and triceps surae muscles in each group 4 months after PC-deletion (PC-del). **B** Weight of isolated muscles 4 months after induction of PC-deletion. Rosa-DTA mice with Tam or NG2-CreERT/DTA without Tam were used as control A and B (Ct A, Ct B), respectively. Values are presented as the means ± SEM (*n *= 4–8); *P < 0.05, **P < 0.01, ns = not significant

**Fig. 6:**
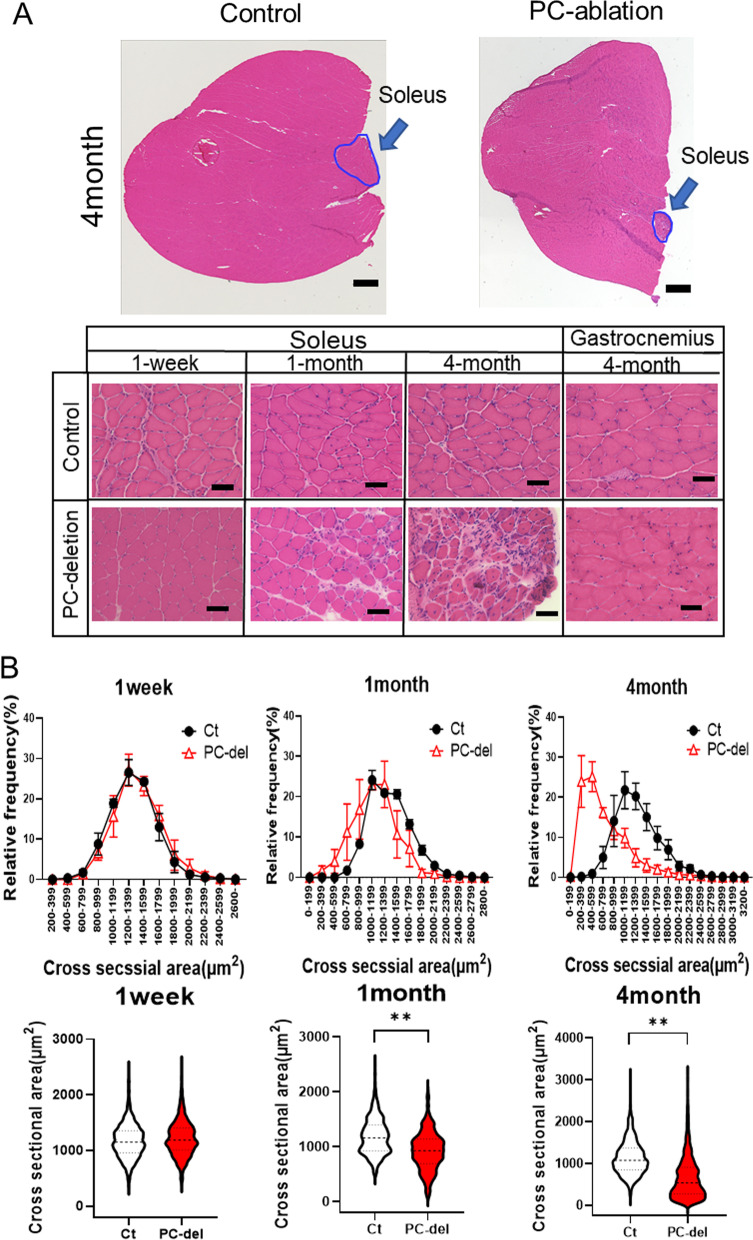
Deletion of NG2^+^ PCs induces atrophy of slow-type myofibers.** A** Hematoxylin–eosin staining short axis view of lower leg muscles at indicated times after induction of NG2^+^ PC-deletion. Scale bar = 500 µm (upper panel) and 50 µm (lower panel). **B** Cross-sectional area (CSA) of soleus muscle fibers calculated at indicated times after induction of PC-deletion (PC-del). NG2-CreERT with Tam was used as a control (Ct). The values are presented as the means ± SEM (*n *= 4–8); **P < 0.01

After 4 months of Tam treatment, the mass of the soleus, a typical slow-type red muscle, was selectively decreased. In contrast, muscular atrophy in fast-type muscles such as the triceps and gastrocnemius was not observed (Fig. 5A, B). Notably, aside from the changes in muscle volume, we also observed alterations in the external characteristics in the soleus, *i.e.,* a whitish appearance; this was not present in other lower limb muscle types (Fig. 5A). Histological analyses indicated significant atrophy of the myofibers in the soleus, but not in the gastrocnemius, after 4 months of Tam treatment (Fig. 6A). Notably, atrophy of myofibers in the soleus muscle was already observed in the first month of Tam treatment. In agreement with the atrophy of the soleus by PC deletion, the cross-sectional area (CSA) of myofibers within the soleus muscle was significantly attenuated. Myofiber CSA distribution across soleus muscles showed a leftward shift owing to the higher abundance of smaller fibers at 1 and 4 months after PC deletion (Fig. 6B).

**Fig. 7:**
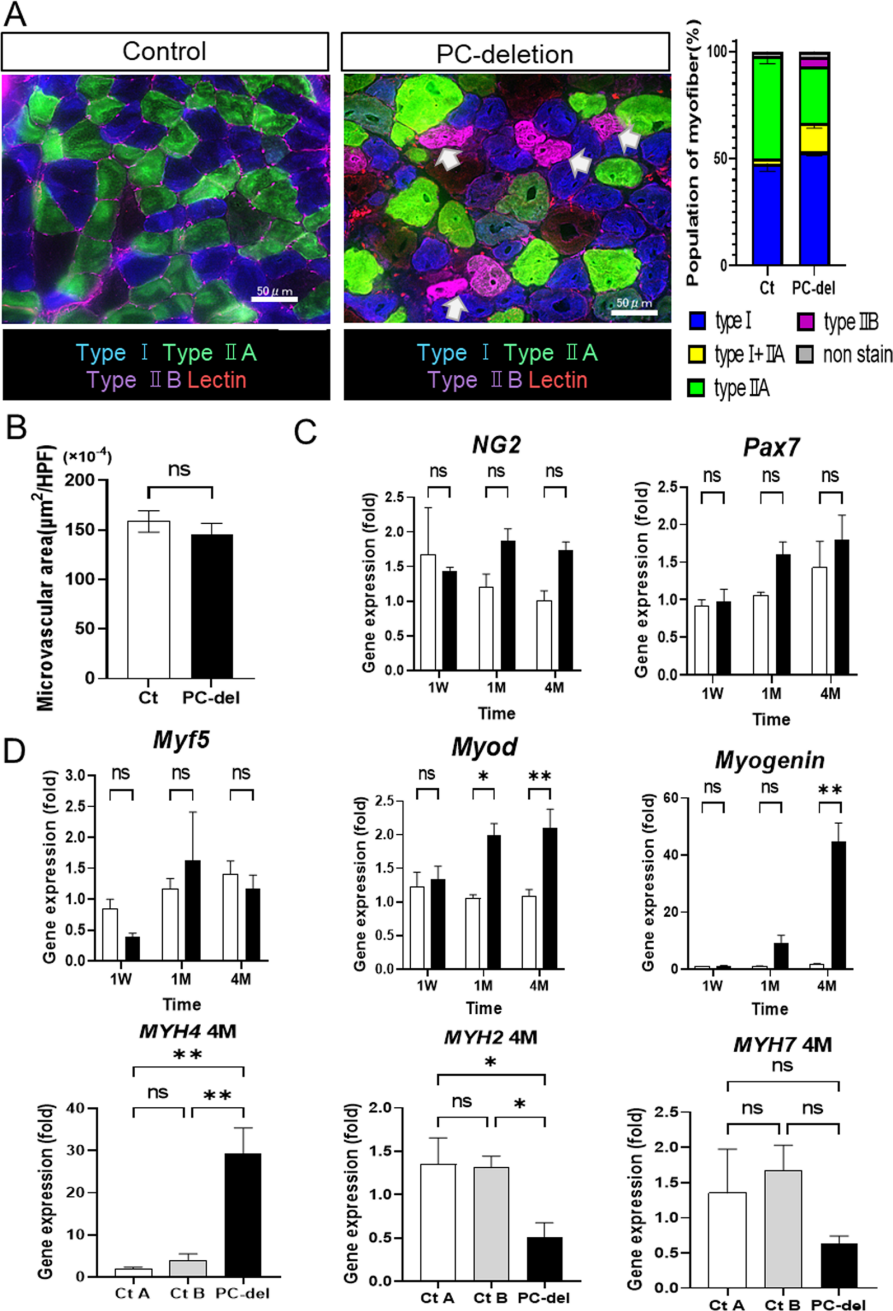
Muscle fiber types and gene expression profiles in soleus after NG2^+^ PC-deletion.** A** At 4 months after PC deletion, the muscle types of each myofiber in the soleus were determined by immunostaining, and the proportion of each myofiber type was calculated (*n *= 4). Scale bar = 50 µm. **B** functional vasculature within muscles was estimated as the area of rhodamine-lectin-stained microvessels per observation area of soleus. Expression of myogenic stem cell marker genes (**C**) and myogenesis-related genes within the soleus muscle (**D**) estimated by quantitative RT-PCR. Closed bars = PC deletion (PC-del), open bars = control (Ct). The values are presented as the means ± SEM (*n *= 3–5); *P < 0.05, **P < 0.01, ns = not significant

The soleus is a muscle that is rich in slow-type muscle fibers (MyHC type I and type IIA fibers) but rare in fast-type muscle fibers (type IIB) (Figs. 1A, 7A). At 4 months after the induction of PC deletion, the proportion of type IIB fibers (fast) was increased and, accordingly, the proportion of type IIA fibers was decreased in the atrophic soleus muscles (Fig. 7A). No differences were observed in lectin-stained functional microvessels within the muscles between the PC-deletion and control groups (Fig. 7B).

### Genomic expression analyses in skeletal muscles under PC-deletion

To confirm the muscular condition under PC deletion, quantitative PCR and comprehensive gene analyses were performed using the soleus muscles of PC-deletion and control mice. PC deletion for 1 month induced a change in gene groups related to several pathways according to Gene Set Enrichment Analysis (GSEA) and Gene Ontology (GO), *e.g., an* increase in inflammation reaction, cell proliferation, and apoptosis, and a decrease in metabolic activity (Table 1, Suppl. Figure 5).

**Table 1:** Comprehensive gene expression profile in response to PC deletion

Group	Symbol	Gene description	Normalized expression		Ratio (fold)	Up/down	
			Control	PC-deletion		Up	Down
Cell marker	CSPG4/NG2	Chondroitin sulfate proteoglycan 4	N.D	15	15.23	***	
	PDGFRβ	Platelet-derived growth factor receptor, beta polypeptide	101	152	1.51		
	Pax7	Paired box 7	N.D	N.D	1.00		
	PDGFRα	Platelet-derived growth factor receptor, alpha polypeptide	310	410	1.32		
	PECAM1	Platelet/endothelial cell adhesion molecule 1	N.D	N.D	0.65		
	ACTA1	Actin, alpha 1, skeletal muscle	36,501	13,449	0.37		*
	ACTA2	Actin, alpha 2, smooth muscle, aorta	387	628	1.63		
Myogenic marker	Myf5	Myogenic factor 5	N.D	13	12.59	***	
	Myod1	Myogenic differentiation 1	35	50	1.43		
	Myogenin	Myogenin	21	403	19.06	***	
	Myf6	Myogenic factor 6	1168	1303	1.12		
	Myh2	Myosin, heavy polypeptide 2, skeletal muscle, adult	26,914	10,276	0.38		*
	Myh3	Myosin, heavy polypeptide 3, skeletal muscle, embryonic	97	1167	12.07	***	
	Myh4	Myosin, heavy polypeptide 4	1515	2887	1.91		
	Myh7	Myosin, heavy polypeptide 7	265	78	0.30		*
Slow myofiber maintenance	NFATc1	Nuclear factor of activated T cells, cytoplasmic, calcineurin dependent 1	37	26	0.69		
	PPARGC1a	Peroxisome proliferative activated receptor, gamma, coactivator 1 alpha	90	58	0.64		
	MEF2a	Myocyte enhancer factor 2A	82	77	0.95		
	MEF2c	Myocyte enhancer factor 2C	883	579	0.66		
	MEF2d	Myocyte enhancer factor 2D	256	121	0.47		*
	FOXO3	Forkhead box O3	152	239	1.57		
Hypoxic	HIF1α	Hypoxia inducible factor 1, alpha subunit	78	163	2.11	*	
	BNIP3	BCL2/adenovirus E1B interacting protein 3	4452	2064	0.46		*
	PDK1	Pyruvate dehydrogenase kinase, isoenzyme 1	207	125	0.60		
	Slc2a1/GLUT1	Solute carrier family 2 (facilitated glucose transporter), member 1	57	62	1.10		
Proliferation/Growth factor	PDGFα	Platelet-derived growth factor, alpha	23	31	1.34		
	PDGFβ	Platelet-derived growth factor, B polypeptide	N.D	11	11.45	***	
	VEGFα	Vascular endothelial growth factor A	114	63	0.55		
	VEGFβ	Vascular endothelial growth factor B	1362	474	0.35		*
	Egf	Epidermal growth factor	87	119	1.37		
	Fgf1	Fibroblast growth factor 1	221	114	0.51		
Apoptosis	Casp2	Caspase 2	10	26	2.47	*	
	Casp3	Caspase 3	21	129	6.07	**	
	Casp6	Caspase 6	30	34	1.12		
	Casp7	Caspase 7	8	28	3.67	*	
	Casp9	Caspase 9	31	12	0.38		*
	Bax	BCL2-associated X protein	78	114	1.46		

After induction of NG2^+^ PC deletion for 1 month, gene expression within the soleus of PC-deletion and control mice was estimated by microarray analysis. The signal for each gene was normalized using the global normalization method. The definition of a significant difference was more or less than twice (up/down * 2, ** 4, *** 8) the difference in the log2 ratio among genes with a fluorescence value of > 100 (boldface). *ND* Not detected

Although we confirmed that Tam treatment induced the deletion of NG2^+^ PCs using the NG2-CreERT/DTA system in in vitro experiments (Additional file 1: Fig S4), observing PC deletion in tissues in vivo was challenging. NG2 gene expression in the soleus muscles was not altered during early (one week) to extended periods (4 months) after the induction of PC deletion (Fig. 7C). The expression of *Pax7* (an SC marker) was also not altered (Fig. 7C). Gene array analysis demonstrated that the expression of PC marker genes, including *PDGFRβ* and smooth muscle actin (*acta2*), or the SC marker *Pax7* did not change, while the expression of *NG2* tended to increase (Table 1).

The deletion of PCs leads to muscle atrophy; thus, the expression of the myogenesis-related genes (*MyoD* and *Myogenin*) was significantly increased in the PC-deletion group compared to the controls (Fig. 7D, Table 1). Furthermore, in line with the alterations in MyHC expression patterns, the expression levels of muscle-type specific myogenic genes, *Myh7* (slow type) and *Myh2* (intermediate type), decreased, while those of *Myh4* (fast type) increased significantly (Fig. 7D).

## Discussion

NG2^+^ PC-specific lineage tracing experiments demonstrated that PCs contribute to myogenesis in adult steady-state conditions, especially slow-type myofibers such as the soleus. Fluorescent tdTomato-expressing PCs fused into multinuclear myofibers and, subsequently, PC-originated nuclei labeled the entire myofiber. In line with the myonuclear domain theory, the number of myonuclei in each myofiber was constant with a fixed muscle volume under homeostatic conditions. In addition, we demonstrated that NG2^+^ PC deletion induced muscular atrophy in a slow-type myofiber-specific manner. This evidence denies the possibility of a non-specific fusion of labeled PCs to myofibers in lineage-tracing experiments. Collectively, these data indicate that the myonuclear turnover of slow-type myofibers is relatively fast, and PCs act as myonuclear suppliers to maintain homeostasis in slow-type myofibers.

### *Discrepancy between *in vivo* and *in vitro* PC-deletion studies*

Because PC is a crucial component of microvessels that regulates and maintains their function and structure, their deletion may cause circulation disorders. It is well documented that genomic deletion or antibody-mediated blocking of PDGFβ, which is a marker for PCs/smooth muscle cells and mediates their function, causes chronic PC deletion and, subsequently, several abnormalities due to microvascular circulation disorder [31, 32]. Alternatively, PDGFRβ^+^-deficient mice exhibit reduced body weight and fail to survive beyond the postnatal first week, presumably due to severe impairment of vessel wall integrity [33, 34]. In contrast to PDGFRβ^+^ PCs, NG2^+^ PC deletion induces a reduction in PC coverage of cortical capillaries at 3 days post-PC deletion and rapid neurovascular uncoupling in the brain circulation system only in the acute phase; the long-term effects of PC deletion have not been reported [35]. Similarly, in the present study, circulation disorders and related phenotypic abnormalities, including body weight loss and muscle atrophy, were not observed (Figs. 4, 7B). In addition, gene expression profiles did not indicate the prevalence of ischemic disorders in PC-deleted muscle tissues, *i.e.,* the angiogenesis-related genes such as *VEGF* and *PECAM1* did not increase. However, the expression of some ischemic-related genes, such as HIF, was slightly elevated (Table 1).

In contrast to the in vivo system discussed above, in vitro experiments using the NG2-CreERT/DTA system demonstrated that Tam treatment induced NG2^+^ PC deletion and, accordingly, the gene expression of NG2 was decreased (Additional file 1: Fig S5). This discrepancy between in vivo and in vitro experiments may be explained by compensatory PC replacement. Berthiaume et al*.* [36] reported that an in vivo laser beam-mediated acute single PC deletion causes temporary loss of microvascular tone at the deletion site; however, PCs were promptly refilled in this area, although little is known about the “PC progenitor cells” responsible for PC-replenishment. NG2 is a specific marker for PCs, whereas PDGFRβ is a relatively wide range PC/smooth muscle cell marker, including presumably PC progenitor cells [10, 37]. Thus, the different phenotype in cell-deletion experiments targeting between NG2^+^ PCs and PDGFRβ^+^ PCs might be owing to the broad range of PC populations. Deletion of relatively broad PC populations, *i.e.,* PDGFRβ^+^ PCs, may abolish the PC compensatory function and induce severe microvascular dysfunction.

Induction of NG2^+^ PC deletion in NG2CreER/DTA mice paradoxically maintained the expression of NG2 genes. This may be due to the balance between the deletion and regeneration of NG2^+^ cells by PC renewal. The compensatory PC regeneration system could maintain the basic circulatory function and structure of microvessels. It is well documented that dysfunction of PCs, namely PC loss followed by microvascular disorder, is a fundamental pathological feature of advanced diabetes mellitus complications [38]. Hyperglycemia induces damage in vascular cells including PCs within hours or days through the excess production of reactive oxygen species (ROS) [39]. A crucial unanswered question is why hyperglycemia in humans in vivo cases requires a decade or more to provoke microvascular disorder followed to diabetes-related complications, such as retinopathy and cardiovascular diseases. The gap of the timing of toxic effects of hyperglycemia in vivo may be explained by the presence of PC-replenishment system. Further studies are required for the mechanisms of compensatory PC-replenishment system.

### Rapid myonuclear turnover in slow-type fibers

In adulthood, skeletal muscle myonuclear number is maintained under steady conditions, with the sporadic fusion of myocytes to compensate for muscle turnover from daily wear and tear [1]. PC-specific lineage tracing experiments have demonstrated that PCs contribute to myonuclear replacement, and myonuclear turnover is relatively fast, at least in slow-type myofibers. This is supported by previous studies demonstrating that the myonuclear turnover of the soleus is higher than that of other fibers, using labeled myonuclear tracing techniques [2]. In mammalian skeletal muscle, multiple myofiber types are intermingled within a single muscle group. Each muscle group exhibits varying proportions of the different fiber types, and muscle fibers can remodel their phenotypes to adapt to environmental changes. Reduced muscle usage from paralysis or prolonged bed rest, namely immobilization and disuse syndrome, causes significant atrophy of all muscles. It is accompanied by a decrease in myonuclear number, particularly in type I fibers compared to type II fibers [1, 2, 40]. From the energy balance perspective, this is reasonable, as the energy consumption of type I fibers is high. Thus, rapid myonuclear turnover might contribute to a quick adaptive response to regulate fiber volume.

In our lineage tracing experiments, PCs were labeled by the expression of tdTomato only once before observation. Skeletal muscle fibers were labeled by tdTomato, and their ratio to total muscle fibers reached a peak (approximately 80–90% in the soleus) within 1 month. Since myonuclear turnover is rapid and labeled PCs are consumed for frequent myonuclear replacement, the proportion of labeled myofibers may decrease unless tdTomato-expressing myonuclei are continuously supplied. However, the proportion of labeled fibers was maintained for more than 1 year. In general, somatic stem cells, including myogenic stem cells, are induced to divide for tissue demands such as muscle damage or increased activation. During the division of cells for myogenic differentiation, it appears that at least one of the daughter cells is maintained as a stem cell, namely asymmetric cell division [41]. According to previous studies [14, 16], PCs act as myogenic stem cells in vivo. Thus, when PCs are labeled as Td-tomato-expressing cells at the start point, labeled PCs continuously supply myonuclei to the fiber syncytium. In contrast, some self-renew and are maintained as labeled PCs. Indeed, in addition to labeled myofibers, labeled PCs were well maintained even after a long observation period of more than 1 year (Figs. 1C, 7A).

Myogenic stem cells, satellite cells derived from fast- or slow-type muscles are heterogeneous cells with different differentiation potentials [42, 43]. In our in vitro myogenesis system, isolated PCs from soleus muscles differentiate to MyHC-positive myofibers (Fig. 3) but not to advanced type specific myofibers (Additional file 1: Fig S4). It remains to seek certain condition to induce further myo-differentiation to confirm PCs have heterogenic myogenic potentials. Alternatively, additional external stimuli are required to induce advanced myofiber differentiation. It has been reported that the fate of SCs during muscle regeneration is primarily influenced by a complex network of intrinsic and also extrinsic regulators such as innervation [44, 45].

### Significance of slow-type muscle specificity

The myofibers switched from type I (slow) to type II (fast) in an adaptive response to reduced myonuclear supplementation from PCs. The percentage of SCs in the soleus muscle is generally higher than that in other muscles [46, 47]; thus, under PC deletion, myogenesis might be mediated by other myogenic stem cells, such as SCs, which can differentiate into fast-type myofibers [48]. However, this compensatory myogenesis is not sufficient to compensate for muscular atrophy that results from PC deletion. The niche surrounding SCs is crucial for regulating their functions and affecting muscle regeneration [6]. PCs also act as SC-associated cells to form a niche for SCs during the neonatal period [19]. Thus, PC deletion might attenuate SC functions for muscular homeostasis even in adulthood, although there was no change in the expression of SC marker genes such as Pax7 (Fig. 7C).

Skeletal muscle fibers vary in their metabolic characteristics, *i.e.,* type I fibers have a highly oxidative metabolism with high capillary density, and type II fibers are further defined as type IIA (oxidative) and type IIB types (having oxidative and glycolytic metabolic characteristics) [30]. In addition to oxidative metabolism, type I slow-twitch myofibers have high lipid oxidative capacity and increased insulin-stimulated glucose uptake, with a high content of insulin-regulating glucose transport protein (GLUT4) compared to type II fibers [49]. The proportion of type I myofibers correlates with insulin responsiveness and may be involved in the etiology and insulin resistance in obesity. Obesity and type 2 diabetes mellitus are associated with reduced proportions of type I fibers and, conversely, increased proportions of type IIB fibers in the skeletal muscle [50, 51]. In our study, the expression profiles of genes related to the generation of metabolites and energy were significantly reduced in the soleus muscle under PC deletion (Additional file 1: Fig S6). Thus, dysfunction of PCs that is a fundamental feature of diabetes mellitus [38] may contribute to the metabolic disorder in diabetes mellitus through the slow-type specific skeletal muscle atrophy, in addition to microvascular disorders. The PC-deletion mouse model could be utilized as a type I-muscle fiber specific atrophy model, and further studies are required to investigate the role of type 1 muscle fibers in the metabolisms and pathogenesis of diabetes mellitus.

## Conclusions

The myonuclear turnover of slow-type muscle fibers is relatively fast. PCs contribute to myonuclear supplementation, acting as myoprogenitor cells for the homeostatic maintenance of type I muscle fibers. The mouse model is valuable for investigating the role of slow-type muscle, especially in metabolism. Thus, understanding the fiber type-specific role may provide critical insights into the pathophysiology of metabolic syndrome, obesity, and type 2 diabetes mellitus and their potential treatments. The mechanisms by which PCs are renewed after their deletion and their interaction with SCs and muscular neurons for the homeostatic maintenance of muscle tissue require further investigation.

### Supplementary Information

**Additional file 1: Fig. S1.** Localization of NG2^+^ cells in adult skeletal muscle tissues. Circulating vessels in NG2-DsRed mice were visualized by intravenous injection of FITC-conjugated lectin. The lower limb skeletal muscles (gastrocnemius and soleus) were fixed and transparentized with RapiClear reagent. Microvessels, lectin-labeled endothelium tubes (lectin; green), and NG2^+^ cells (DsRed; red) within transparent muscles were visualized in a 3D view using confocal fluorescent microscopy. The nuclei were counterstained with DAPI. Scale bar = 50 µm. **Fig. S2.** NG2^+^ cell lineage tracing within the skeletal muscle. The tdTomato expression driven by the universal Rosa26 promoter was specifically induced in NG2^+^ cells using NG2-CreERT/Rosa-tdTomato mice. After five days of constitutive treatment with Tam, NG2^+^ cells were expressed. B. On day 1 of the observation period, tdTomato^+^ cells were observed only at perivascular sites, such as PCs. On day 21, tdTomato-expressing myofibers were observed in most muscle tissues. The ratio of tdTomato^+^ myofibers to total myofibers varied by muscle site, i.e., over 80% of tdTomato^+^ myofibers in the soleus and diaphragm and 20–30% in the gastrocnemius and rectus abdominal muscles. Scale bar = 200 µm. **Fig. S3**. Schematic diagram for the *in vitro* muscular differentiation assay. Myofibers were isolated from the soleus of NG2-CreERT/Rosa-tdTomato mice by using a collagenase-containing medium. Isolated myofibers were incubated in DMEM-containing 10% FBS and Tam (2 µM) for three days to label NG2^+^ PCs. The medium was then changed to a differentiation medium containing 2% horse serum. After six days of induction, the myogenesis of NG2^+^ PCs was observed. **Fig. S4.** *In vitro* myogenic potency of NG2^+^ PCs from soleus muscles. A. Myofibers isolated from the soleus of NG2-CreERT/Rosa-tdTomato mice, which were incubated in DMEM-containing hydroxy tamoxifen (Tam) for three days to label NG2^+^ PCs. After six days of differentiation induction, myogenesis was determined by immunostaining with myosin heavy chain (MyHC) and myosin heavy chain (MYH) isoform 2 and 7. Isolated soleus myofibers were used for control for immunostaining. Scale bars = 100 µm. **Fig. S5.** Tam treatment induces deletion of NG2^+^ cells. A. *In vitro* effects of 4-hydroxytamoxifen (4-HT) on NG2^+^ cells isolated from subcutaneous adipose tissues of NG2-CreERT/Rosa26-DTA mice. Cells at confluent were incubated in medium containing hydroxy-Tam for 5 days, and the number of cells were counted. B. Gene expression of NG2 in NG2^+^ cells with Tam was esteemed by qPCR. **Fig. S6.** Microarray enrichment analysis in response to PC deletion. After induction of NG2^+^ PC deletion for one month, microarray analysis of the soleus of PC-deletion and control mice was performed. The top 20 upregulated and downregulated pathway-related gene sets are listed.

## Data Availability

The data in this study were generated at Asahikawa Medical University and available from the corresponding author upon reasonable request. The microarray data will be available in GEO entitled Gene expression changes in soleus muscle in mice after pericyte-deletion. Accession number of microarray is GSE229384 for GEO accession (https://www.ncbi.nlm.nih.gov/geo/query/acc.cgi?acc=GSE229364).

## Abbreviations

PCs: Pericytes
SCs: Satellite cells
ASCs: Adipose stromal cells
MSCs: Mesenchymal stem cells
FAPs: Fibro-adipogenic progenitors
SPF: Specific pathogen-free
Tam: Tamoxifen
DTA: Diphtheria toxin A
FBS: Fetal bovine serum
PBS: Phosphate buffered saline
FITC: Fluorescein isothiocyanate
PFA: Paraformaldehyde
NG2: Neuroglial 2 proteoglycan, Cspg4
Pax: Paired box
Myf: Myogenic factor
Myh: Myosin heavy chain
MyoD: Myogenic differentiation
GAPDH: Glyceraldehyde-3-phosphate dehydrogenase
PDGFR: Platelet-derived growth factor receptor
FISH: Fluorescence in situ hybridization
