# Supplementary material for: NG2-positive pericytes regulate homeostatic maintenance of slow-type skeletal muscle with rapid myonuclear turnover
Source: Stem Cell Res Ther. 2023 Aug 17;14:205. doi: 10.1186/s13287-023-03433-1 (PMC10433572; doi:10.1186/s13287-023-03433-1)
Supplement: Supplementary file 1 — Additional file 1: Fig. S1. Localization of NG2+ cells in adult skeletal muscle tissues. Circulating vessels in NG2-DsRed mice were visualized by intravenous injection of FITC-conjugated lectin. The lower limb skeletal muscles (gastrocnemius and soleus) were fixed and transparentized with RapiClear reagent. Microvessels, lectin-labeled endothelium tubes (lectin; green), and NG2+ cells (DsRed; red) within transparent muscles were visualized in a 3D view using confocal fluorescent microscopy. The nuclei were counterstained with DAPI. Scale bar = 50 µm. Fig. S2. NG2+ cell lineage tracing within the skeletal muscle. The tdTomato expression driven by the universal Rosa26 promoter was specifically induced in NG2+ cells using NG2-CreERT/Rosa-tdTomato mice. After five days of constitutive treatment with Tam, NG2+ cells were expressed. B. On day 1 of the observation period, tdTomato+ cells were observed only at perivascular sites, such as PCs. On day 21, tdTomato-expressing myofibers were observed in most muscle tissues. The ratio of tdTomato+ myofibers to total myofibers varied by muscle site, i.e., over 80% of tdTomato+ myofibers in the soleus and diaphragm and 20–30% in the gastrocnemius and rectus abdominal muscles. Scale bar = 200 µm. Fig. S3. Schematic diagram for the in vitro muscular differentiation assay. Myofibers were isolated from the soleus of NG2-CreERT/Rosa-tdTomato mice by using a collagenase-containing medium. Isolated myofibers were incubated in DMEM-containing 10% FBS and Tam (2 µM) for three days to label NG2+ PCs. The medium was then changed to a differentiation medium containing 2% horse serum. After six days of induction, the myogenesis of NG2+ PCs was observed. Fig. S4. In vitro myogenic potency of NG2+ PCs from soleus muscles. A. Myofibers isolated from the soleus of NG2-CreERT/Rosa-tdTomato mice, which were incubated in DMEM-containing hydroxy tamoxifen (Tam) for three days to label NG2+ PCs. After six days of differentiation induction, myogenesis w [file 13287_2023_3433_MOESM1_ESM.docx]

**Supporting Information**

**NG2-positive pericytes regulate homeostatic maintenance of slow-type skeletal muscle with rapid myonuclear turnover**

**Takamitsu Tatsukawa et al.**

**
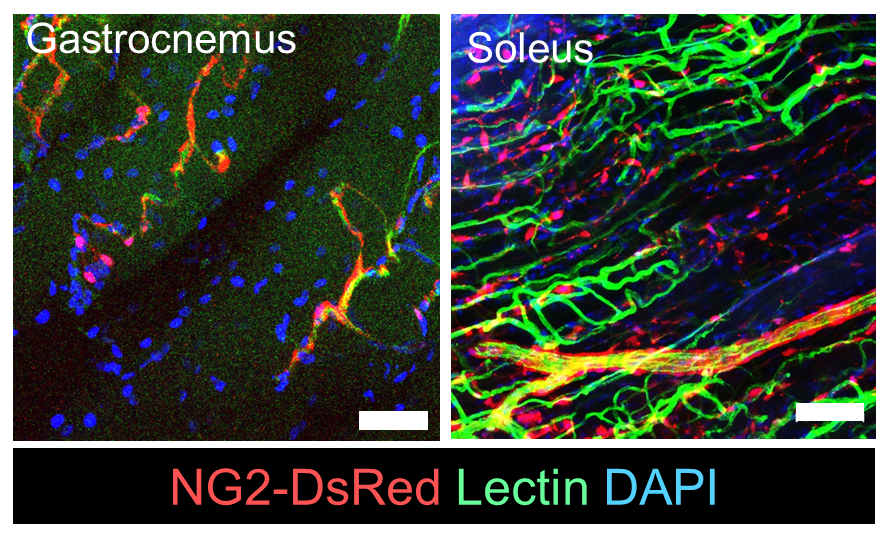
**

**Supplementary Figure 1. Localization of NG2^+^ cells in adult skeletal muscle tissues.** Circulating vessels in NG2-DsRed mice were visualized by intravenous injection of FITC-conjugated lectin. The lower limb skeletal muscles (gastrocnemius and soleus) were fixed and transparentized with RapiClear reagent. Microvessels, lectin-labeled endothelium tubes (lectin; green), and NG2^+^ cells (DsRed; red) within transparent muscles were visualized in a 3D view using confocal fluorescent microscopy. The nuclei were counterstained with DAPI. Scale bar = 50 μm.


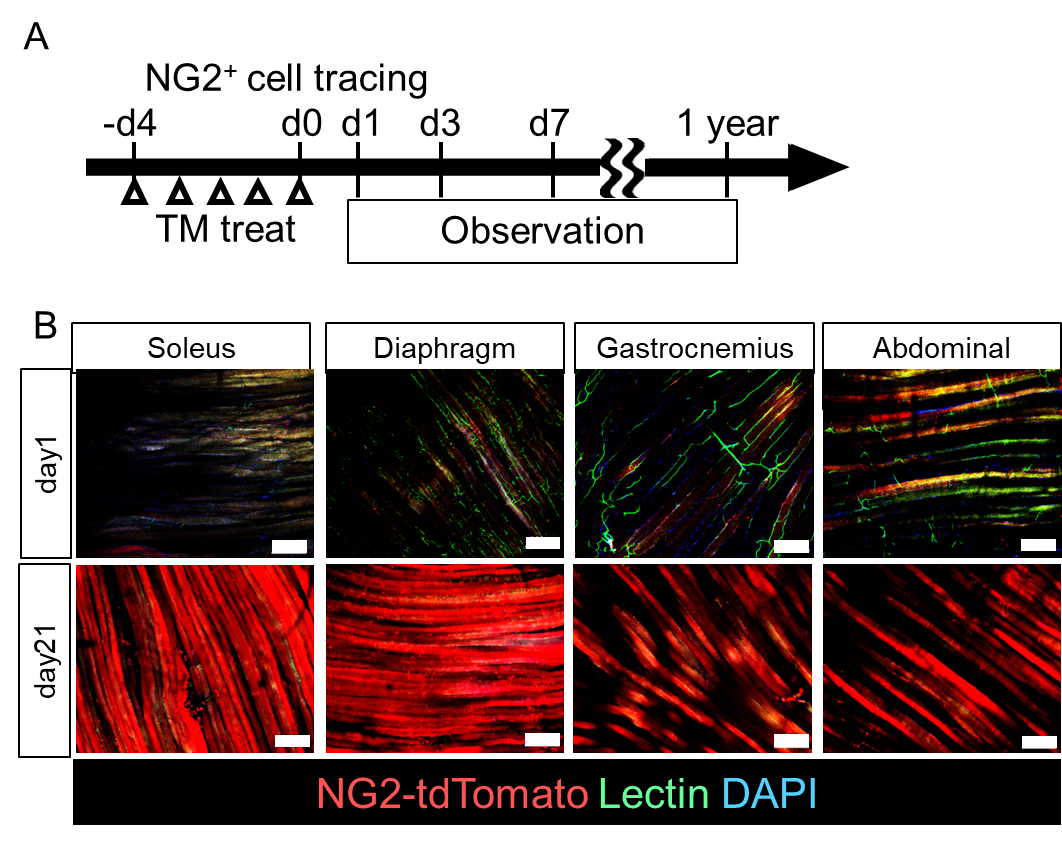


**Supplementary Figure 2. NG2^+^ cell lineage tracing within the skeletal muscle.** The tdTomato expression driven by the universal Rosa26 promoter was specifically induced in NG2^+^ cells using NG2-CreERT/Rosa-tdTomato mice. After five days of constitutive treatment with Tam, NG2^+^ cells were expressed. **B.** On day 1 of the observation period, tdTomato^+^ cells were observed only at perivascular sites, such as PCs. On day 21, tdTomato-expressing myofibers were observed in most muscle tissues. The ratio of tdTomato^+^ myofibers to total myofibers varied by muscle site, *i.e.,* over 80% of tdTomato^+^ myofibers in the soleus and diaphragm and 20–30% in the gastrocnemius and rectus abdominal muscles. Scale bar = 200 μm.


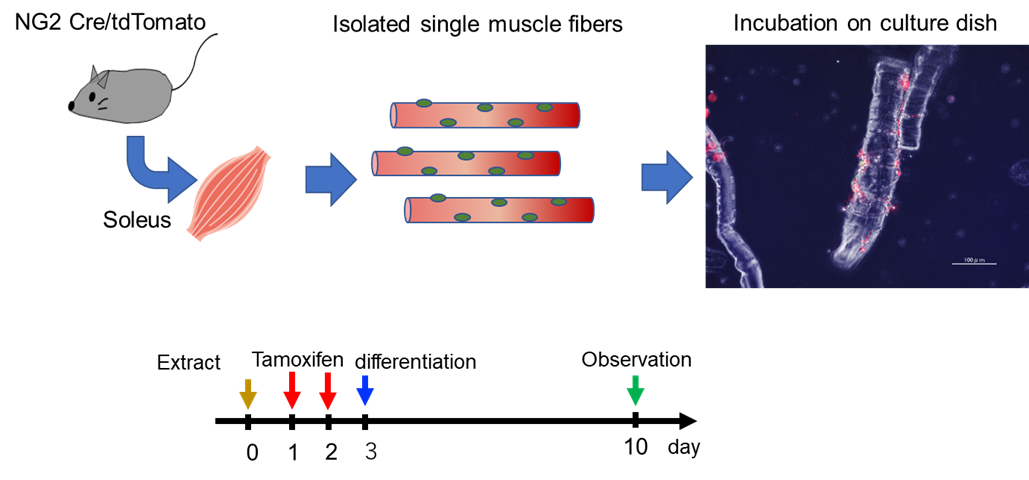


**Supplementary Figure 3. Schematic diagram for the *in vitro* muscular differentiation assay.** Myofibers were isolated from the soleus of NG2-CreERT/Rosa-tdTomato mice by using a collagenase-containing medium. Isolated myofibers were incubated in DMEM-containing 10% FBS and Tam (2 µM) for three days to label NG2^+^ PCs. The medium was then changed to a differentiation medium containing 2% horse serum. After six days of induction, the myogenesis of NG2^+^ PCs was observed.


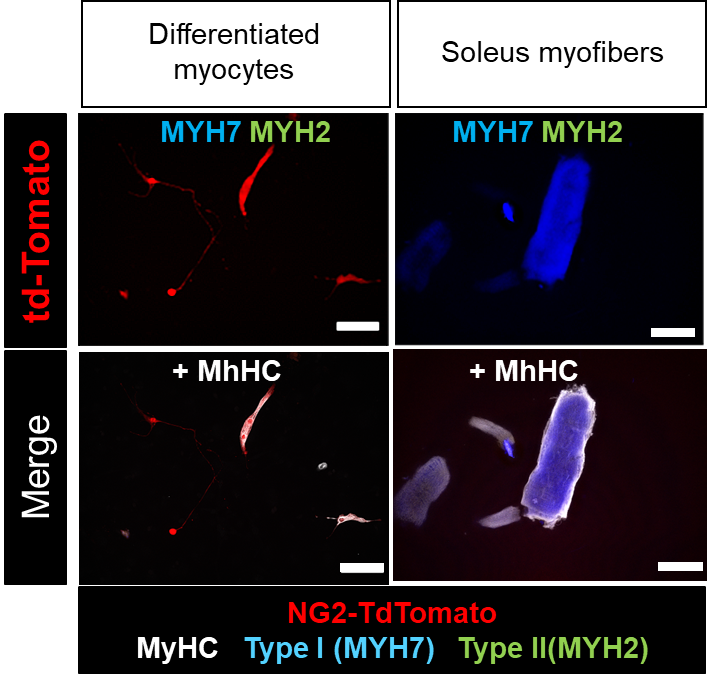


**Supplementary Figure 4. *In vitro* myogenic potency of NG2^+^ PCs from soleus muscles. A.** Myofibers isolated from the soleus of NG2-CreERT/Rosa-tdTomato mice, which were incubated in DMEM-containing hydroxy tamoxifen (Tam) for three days to label NG2^+^ PCs. After six days of differentiation induction, myogenesis was determined by immunostaining with myosin heavy chain (MyHC) and myosin heavy chain (MYH) isoform 2 and 7. Isolated soleus myofibers were used for control for immunostaining. Scale bars = 100 μm.


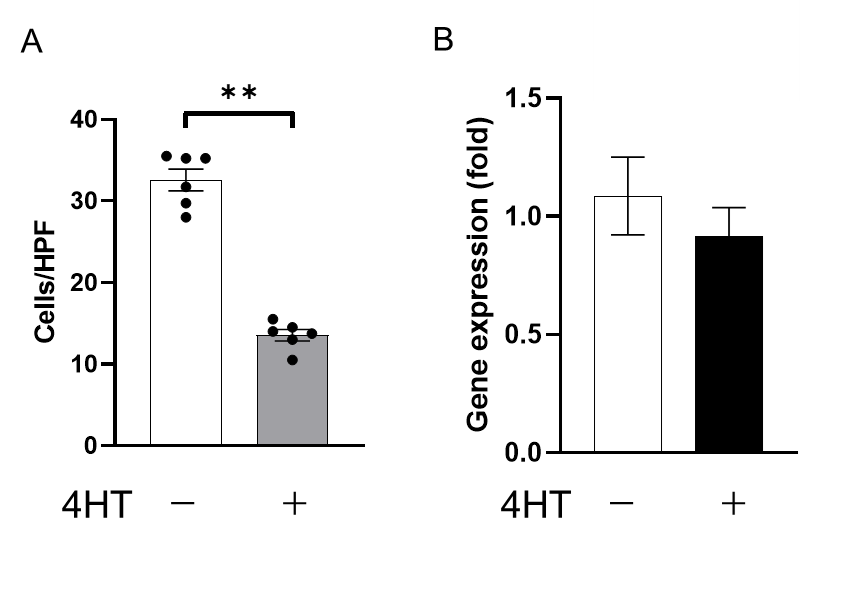


**Supplementary Figure 5. Tam treatment induces deletion of NG2^+^ cells. A.** *In vitro* effects of 4-hydroxytamoxifen (4-HT) on NG2^+^ cells isolated from subcutaneous adipose tissues of NG2-CreERT/Rosa26-DTA mice. Cells at confluent were incubated in medium containing hydroxy-Tam for 5 days, and the number of cells were counted. **B.** Gene expression of NG2 in NG2^+^ cells with Tam was esteemed by qPCR.

**
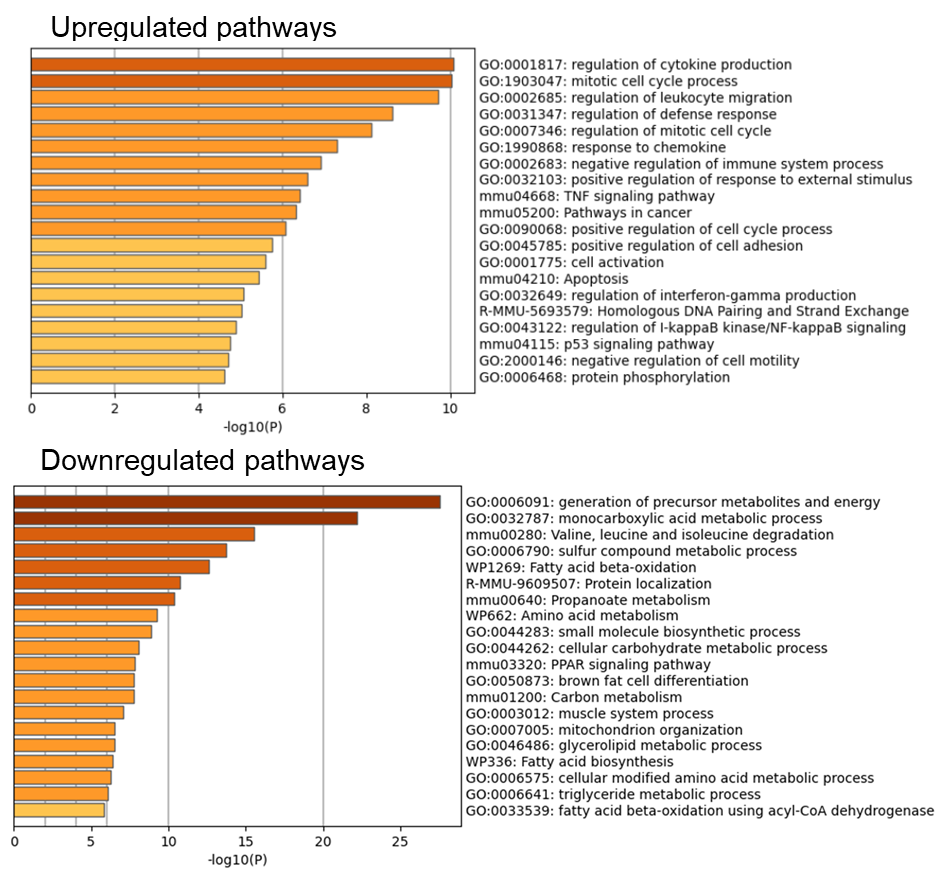
**

**Supplementary Figure 6. Microarray enrichment analysis in response to PC deletion.** After induction of NG2^+^ PC deletion for one month, microarray analysis of the soleus of PC-deletion and control mice was performed. The top 20 upregulated and downregulated pathway-related gene sets are listed.
